# 1,4-Bis[(1*H*-pyrazol-1-yl)meth­yl]benzene dihydrate

**DOI:** 10.1107/S1600536809007521

**Published:** 2009-03-06

**Authors:** Ai-E Shi, Yan-Jun Hou, Yi-Ming Zhang, Guang-Feng Hou, Jin-Sheng Gao

**Affiliations:** a College of Chemistry and Materials Science, Heilongjiang University, Harbin 150080, People’s Republic of China

## Abstract

The asymmetric unit of the title compound, C_14_H_14_N_4_·2H_2_O consists of two half-mol­ecules of the main mol­ecule, each situated on an inversion center, and two mol­ecules of water. One-dimensional chains of water mol­ecules are built up by O—H⋯O hydrogen bonds which are then linked with the main mol­ecule *via* O—H⋯N hydrogen bonds, forming a two-dimensional supra­molecular network in the *ac* plane.

## Related literature

For background and the synthesis, see: Chang *et al.* (1993 ▶). For similar structures, see: Bourne *et al.* (2006 ▶).
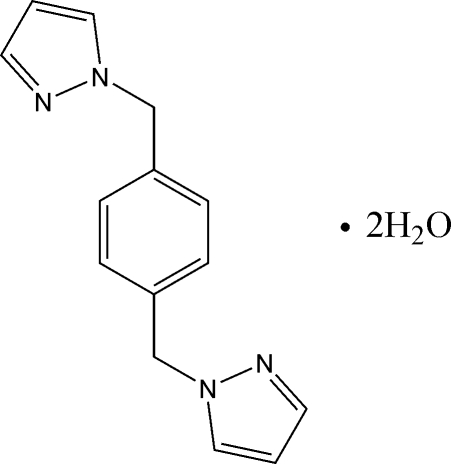


## Experimental

### 

#### Crystal data

C_14_H_14_N_4_·2H_2_O
*M* *_r_* = 274.32
Monoclinic, 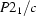

*a* = 4.680 (2) Å
*b* = 18.640 (8) Å
*c* = 16.974 (10) Å
β = 91.15 (2)°
*V* = 1480.6 (13) Å^3^
*Z* = 4
Mo *K*α radiation
μ = 0.09 mm^−1^
*T* = 291 K
0.29 × 0.27 × 0.19 mm

#### Data collection

Rigaku R-AXIS RAPID diffractometer
Absorption correction: multi-scan (*ABSCOR*; Higashi, 1995 ▶) *T*_min_ = 0.956, *T*_max_ = 0.984
14065 measured reflections
3361 independent reflections
1735 reflections with *I* > 2σ(*I*)
*R*_int_ = 0.050

#### Refinement

*R*[*F* ^2^ > 2σ(*F* ^2^)] = 0.048
*wR*(*F* ^2^) = 0.123
*S* = 1.00
3361 reflections
181 parameters
H-atom parameters constrained
Δρ_max_ = 0.14 e Å^−3^
Δρ_min_ = −0.13 e Å^−3^

### 

Data collection: *RAPID-AUTO* (Rigaku, 1998 ▶); cell refinement: *RAPID-AUTO*; data reduction: *CrystalStructure* (Rigaku/MSC, 2002 ▶); program(s) used to solve structure: *SHELXS97* (Sheldrick, 2008 ▶); program(s) used to refine structure: *SHELXL97* (Sheldrick, 2008 ▶); molecular graphics: *SHELXTL* (Sheldrick, 2008 ▶); software used to prepare material for publication: *SHELXL97*.

## Supplementary Material

Crystal structure: contains datablocks global, I. DOI: 10.1107/S1600536809007521/fl2236sup1.cif

Structure factors: contains datablocks I. DOI: 10.1107/S1600536809007521/fl2236Isup2.hkl

Additional supplementary materials:  crystallographic information; 3D view; checkCIF report

## Figures and Tables

**Table 1:** Hydrogen-bond geometry (Å, °)

*D*—H⋯*A*	*D*—H	H⋯*A*	*D*⋯*A*	*D*—H⋯*A*
O1—H15⋯N4	0.85	2.08	2.929 (3)	178
O1—H16⋯O2^i^	0.85	1.87	2.717 (2)	177
O2—H17⋯N2^ii^	0.85	2.08	2.923 (2)	170
O2—H18⋯O1	0.85	1.89	2.733 (2)	172

Symmetry codes: (i) 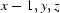; (ii) 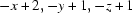.

**Fig. 1.**
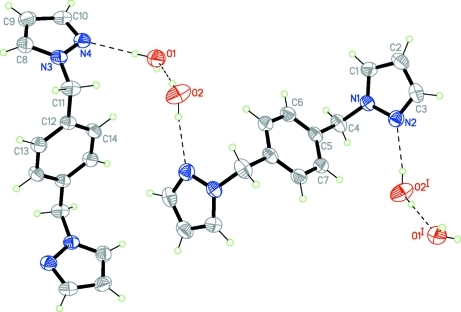
The molecular structure of (I), showing displacement ellipsoids at the 30% probability level for non-H atoms. Dashed lines indicate the hydrogen-bonding interactions [Symmetry codes; (I) -x + 2, -y + 1, -z + 1]

**Fig. 2.**
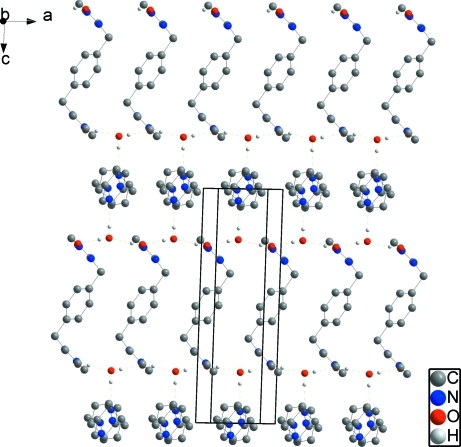
A partial packing view, showing the two-dimensional network. Dashed lines indicate the hydrogen-bonding interactions. Only H atoms involved in hydrogren bonds are shown.

## supplementary crystallographic information

### Comment

The title compound is not only an excellent flexible ligand, but also a hydrogen bonding acceptor that can be used to construct supramolecular structures (Chang *et al.* (1993). In this paper, we report a two-dimensional supramolecular network similar to that reported earlier (Bourne *et al.* 2006), composed of 1,4-bis((1*H*-pyrazol-1-yl)methyl)benzene and water.

In (I), all bond lengths and angles are normal. The flexible ligand molecules display a '*Z*' shape, with the pyrazole rings on opposite sides of the plane of the phenyl ring (Fig. 1).

In the crystal, one-dimensional water chains are built up by O—H···O hydrogen bonding interactions. The chains are then linked to the ligands (I),*via* O—H···N hydrogen bonds, forming a two-dimensional supramolecular network along the *ac* plane (Fig. 2, Table 1).

### Experimental

The title compound was prepared from pyrazole (6.8 g, 100 mmol), Na_2_CO_3_ (16 g, 100 mmol) and 1,4-bis(bromomethyl)benzene (21.3 g, 50 mmol)in benzene. The solution was refluxed for 3 h. The title compound (4.7 g, 20 mmol) was then dissolved in hot water (30 ml) to give a clear solution and allowed to stand in a desiccator at room temperature for several days after which colorless crystals of (I) were obtained.

### Refinement

H atoms bound to C atoms were placed in calculated positions and treated as riding on their parent atoms, with C—H = 0.93 Å (aromatic), C—H = 0.97 Å (methylene), and with *U*_iso_(H) = 1.2*U*_eq_(C). Water H atoms were initially located in a difference Fourier map, but they were treated as riding on their parent atoms with O—H = 0.85 Å and with with *U*_iso_(H) = 1.5*U*_eq_(O).

### Crystal data

**Table:**

C_14_H_14_N_4_·2H_2_O	*F*(000) = 584
*M**_r_* = 274.32	*D*_x_ = 1.231 Mg m^−^^3^
Monoclinic, *P*2_1_/*c*	Mo *K*α radiation, λ = 0.71073 Å
Hall symbol: -P 2ybc	Cell parameters from 8012 reflections
*a* = 4.680 (2) Å	θ = 6.5–54.9°
*b* = 18.640 (8) Å	µ = 0.09 mm^−^^1^
*c* = 16.974 (10) Å	*T* = 291 K
β = 91.15 (2)°	Block, colorless
*V* = 1480.6 (13) Å^3^	0.29 × 0.27 × 0.19 mm
*Z* = 4	

### Data collection

**Table:**

Rigaku R-AXIS RAPID diffractometer	3361 independent reflections
Radiation source: fine-focus sealed tube	1735 reflections with *I* > 2σ(*I*)
graphite	*R*_int_ = 0.050
ω scan	θ_max_ = 27.5°, θ_min_ = 3.3°
Absorption correction: multi-scan (*ABSCOR*; Higashi, 1995)	*h* = −6→5
*T*_min_ = 0.956, *T*_max_ = 0.984	*k* = −23→24
14065 measured reflections	*l* = −22→22

### Refinement

**Table:**

Refinement on *F*^2^	Primary atom site location: structure-invariant direct methods
Least-squares matrix: full	Secondary atom site location: difference Fourier map
*R*[*F*^2^ > 2σ(*F*^2^)] = 0.048	Hydrogen site location: inferred from neighbouring sites
*wR*(*F*^2^) = 0.123	H-atom parameters constrained
*S* = 1.00	*w* = 1/[σ^2^(*F*_o_^2^) + (0.0547*P*)^2^] where *P* = (*F*_o_^2^ + 2*F*_c_^2^)/3
3361 reflections	(Δ/σ)_max_ < 0.001
181 parameters	Δρ_max_ = 0.14 e Å^−^^3^
0 restraints	Δρ_min_ = −0.13 e Å^−^^3^

### Special details

**Table:**

Geometry. All e.s.d.'s (except the e.s.d. in the dihedral angle between two l.s. planes) are estimated using the full covariance matrix. The cell e.s.d.'s are taken into account individually in the estimation of e.s.d.'s in distances, angles and torsion angles; correlations between e.s.d.'s in cell parameters are only used when they are defined by crystal symmetry. An approximate (isotropic) treatment of cell e.s.d.'s is used for estimating e.s.d.'s involving l.s. planes.
Refinement. Refinement of *F*^2^ against ALL reflections. The weighted *R*-factor *wR* and goodness of fit *S* are based on *F*^2^, conventional *R*-factors *R* are based on *F*, with *F* set to zero for negative *F*^2^. The threshold expression of *F*^2^ > σ(*F*^2^) is used only for calculating *R*-factors(gt) *etc*. and is not relevant to the choice of reflections for refinement. *R*-factors based on *F*^2^ are statistically about twice as large as those based on *F*, and *R*- factors based on ALL data will be even larger.

### Fractional atomic coordinates and isotropic or equivalent isotropic displacement parameters (Å^2^)

**Table:**

	*x*	*y*	*z*	*U*_iso_*/*U*_eq_	
C1	0.8625 (5)	0.35411 (10)	0.69781 (13)	0.0727 (6)	
H1	0.7584	0.3209	0.6680	0.087*	
C2	1.0682 (5)	0.33893 (11)	0.75233 (13)	0.0784 (6)	
H2	1.1345	0.2939	0.7677	0.094*	
C3	1.1572 (4)	0.40427 (12)	0.77993 (12)	0.0737 (6)	
H3	1.2983	0.4104	0.8187	0.088*	
C4	0.6675 (4)	0.46818 (11)	0.63845 (12)	0.0709 (6)	
H4	0.6093	0.5124	0.6635	0.085*	
H5	0.4963	0.4419	0.6231	0.085*	
C5	0.8366 (3)	0.48549 (9)	0.56601 (11)	0.0551 (4)	
C6	0.8351 (4)	0.43987 (10)	0.50232 (12)	0.0642 (5)	
H6	0.7232	0.3987	0.5034	0.077*	
C7	1.0049 (4)	0.54625 (9)	0.56293 (11)	0.0622 (5)	
H7	1.0099	0.5779	0.6053	0.075*	
C8	0.5827 (4)	0.27680 (10)	−0.07594 (13)	0.0724 (6)	
H8	0.6642	0.2909	−0.1230	0.087*	
C9	0.3835 (5)	0.22438 (10)	−0.06711 (15)	0.0776 (6)	
H9	0.3016	0.1955	−0.1061	0.093*	
C10	0.3302 (4)	0.22347 (9)	0.01191 (14)	0.0706 (6)	
H10	0.2015	0.1925	0.0354	0.085*	
C11	0.8187 (4)	0.36625 (9)	0.01523 (13)	0.0663 (5)	
H11	0.9808	0.3674	−0.0195	0.080*	
H12	0.8916	0.3612	0.0688	0.080*	
C12	0.6550 (3)	0.43624 (8)	0.00771 (11)	0.0527 (4)	
C13	0.6725 (4)	0.47764 (9)	−0.05894 (11)	0.0616 (5)	
H13	0.7898	0.4630	−0.0995	0.074*	
C14	0.4809 (4)	0.45945 (9)	0.06678 (11)	0.0624 (5)	
H14	0.4663	0.4324	0.1126	0.075*	
N1	0.8358 (3)	0.42535 (8)	0.69450 (8)	0.0559 (4)	
N2	1.0191 (3)	0.45779 (8)	0.74505 (9)	0.0660 (4)	
N3	0.6401 (3)	0.30445 (7)	−0.00459 (10)	0.0579 (4)	
N4	0.4843 (3)	0.27223 (7)	0.05120 (10)	0.0638 (4)	
O1	0.4928 (3)	0.31462 (7)	0.21740 (9)	0.0847 (4)	
H15	0.4939	0.3031	0.1690	0.127*	
H16	0.3374	0.3373	0.2238	0.127*	
O2	0.9966 (3)	0.38581 (8)	0.24291 (10)	0.1062 (6)	
H17	0.9746	0.4311	0.2421	0.159*	
H18	0.8329	0.3663	0.2386	0.159*	

### Atomic displacement parameters (Å^2^)

**Table:**

	*U*^11^	*U*^22^	*U*^33^	*U*^12^	*U*^13^	*U*^23^
C1	0.0920 (14)	0.0568 (11)	0.0693 (14)	−0.0064 (10)	0.0027 (12)	0.0032 (10)
C2	0.0967 (15)	0.0713 (13)	0.0674 (14)	0.0235 (12)	0.0093 (12)	0.0138 (11)
C3	0.0689 (12)	0.0959 (16)	0.0562 (12)	0.0135 (12)	−0.0021 (10)	0.0092 (11)
C4	0.0529 (10)	0.0895 (13)	0.0704 (14)	0.0117 (9)	0.0054 (10)	0.0187 (11)
C5	0.0413 (9)	0.0633 (10)	0.0605 (12)	0.0094 (8)	−0.0013 (8)	0.0145 (9)
C6	0.0646 (11)	0.0599 (10)	0.0679 (13)	−0.0089 (9)	−0.0060 (10)	0.0122 (10)
C7	0.0708 (11)	0.0571 (10)	0.0587 (12)	0.0052 (9)	−0.0026 (10)	0.0019 (9)
C8	0.0854 (14)	0.0656 (12)	0.0664 (15)	0.0127 (11)	0.0027 (11)	0.0025 (10)
C9	0.0921 (15)	0.0559 (11)	0.0839 (18)	0.0042 (11)	−0.0177 (13)	−0.0075 (11)
C10	0.0695 (12)	0.0487 (10)	0.0935 (18)	−0.0009 (9)	0.0001 (12)	0.0009 (10)
C11	0.0505 (10)	0.0544 (10)	0.0936 (16)	0.0012 (8)	−0.0073 (10)	0.0040 (10)
C12	0.0423 (8)	0.0491 (9)	0.0665 (12)	−0.0049 (7)	−0.0047 (8)	0.0034 (8)
C13	0.0599 (10)	0.0607 (11)	0.0646 (13)	0.0022 (8)	0.0130 (9)	0.0007 (9)
C14	0.0708 (11)	0.0570 (10)	0.0596 (12)	0.0019 (9)	0.0029 (10)	0.0135 (9)
N1	0.0495 (8)	0.0641 (9)	0.0543 (9)	0.0008 (7)	0.0061 (7)	0.0091 (7)
N2	0.0680 (9)	0.0699 (9)	0.0601 (10)	−0.0009 (8)	0.0042 (8)	−0.0023 (8)
N3	0.0561 (8)	0.0491 (7)	0.0682 (11)	0.0080 (7)	−0.0015 (8)	0.0022 (8)
N4	0.0669 (9)	0.0537 (8)	0.0709 (11)	0.0047 (8)	0.0043 (8)	0.0025 (8)
O1	0.0878 (9)	0.0774 (9)	0.0887 (11)	−0.0004 (7)	−0.0012 (8)	0.0027 (8)
O2	0.0867 (10)	0.0885 (10)	0.1433 (16)	0.0021 (8)	0.0031 (10)	−0.0361 (10)

### Geometric parameters (Å, °)

**Table:**

C1—N1	1.335 (2)	C9—C10	1.369 (3)
C1—C2	1.352 (3)	C9—H9	0.9300
C1—H1	0.9300	C10—N4	1.331 (2)
C2—C3	1.367 (3)	C10—H10	0.9300
C2—H2	0.9300	C11—N3	1.459 (2)
C3—N2	1.322 (2)	C11—C12	1.517 (2)
C3—H3	0.9300	C11—H11	0.9700
C4—N1	1.460 (2)	C11—H12	0.9700
C4—C5	1.510 (2)	C12—C13	1.373 (2)
C4—H4	0.9700	C12—C14	1.375 (2)
C4—H5	0.9700	C13—C14^ii^	1.380 (2)
C5—C6	1.375 (3)	C13—H13	0.9300
C5—C7	1.381 (2)	C14—C13^ii^	1.380 (2)
C6—C7^i^	1.374 (3)	C14—H14	0.9300
C6—H6	0.9300	N1—N2	1.345 (2)
C7—C6^i^	1.374 (3)	N3—N4	1.348 (2)
C7—H7	0.9300	O1—H15	0.8500
C8—N3	1.339 (2)	O1—H16	0.8500
C8—C9	1.361 (3)	O2—H17	0.8500
C8—H8	0.9300	O2—H18	0.8500
			
N1—C1—C2	107.56 (18)	C10—C9—H9	127.6
N1—C1—H1	126.2	N4—C10—C9	112.01 (18)
C2—C1—H1	126.2	N4—C10—H10	124.0
C1—C2—C3	104.84 (18)	C9—C10—H10	124.0
C1—C2—H2	127.6	N3—C11—C12	111.94 (14)
C3—C2—H2	127.6	N3—C11—H11	109.2
N2—C3—C2	112.08 (19)	C12—C11—H11	109.2
N2—C3—H3	124.0	N3—C11—H12	109.2
C2—C3—H3	124.0	C12—C11—H12	109.2
N1—C4—C5	111.25 (14)	H11—C11—H12	107.9
N1—C4—H4	109.4	C13—C12—C14	118.04 (16)
C5—C4—H4	109.4	C13—C12—C11	120.95 (17)
N1—C4—H5	109.4	C14—C12—C11	121.00 (16)
C5—C4—H5	109.4	C12—C13—C14^ii^	121.16 (16)
H4—C4—H5	108.0	C12—C13—H13	119.4
C6—C5—C7	118.11 (16)	C14^ii^—C13—H13	119.4
C6—C5—C4	120.89 (17)	C12—C14—C13^ii^	120.80 (16)
C7—C5—C4	120.97 (18)	C12—C14—H14	119.6
C7^i^—C6—C5	121.54 (17)	C13^ii^—C14—H14	119.6
C7^i^—C6—H6	119.2	C1—N1—N2	111.26 (16)
C5—C6—H6	119.2	C1—N1—C4	128.31 (17)
C6^i^—C7—C5	120.36 (17)	N2—N1—C4	119.92 (15)
C6^i^—C7—H7	119.8	C3—N2—N1	104.26 (16)
C5—C7—H7	119.8	C8—N3—N4	111.25 (16)
N3—C8—C9	107.6 (2)	C8—N3—C11	128.06 (17)
N3—C8—H8	126.2	N4—N3—C11	120.37 (16)
C9—C8—H8	126.2	C10—N4—N3	104.34 (16)
C8—C9—C10	104.80 (19)	H15—O1—H16	105.7
C8—C9—H9	127.6	H17—O2—H18	108.3

Symmetry codes: (i) −*x*+2, −*y*+1, −*z*+1; (ii) −*x*+1, −*y*+1, −*z*.

### Hydrogen-bond geometry (Å, °)

**Table:**

*D*—H···*A*	*D*—H	H···*A*	*D*···*A*	*D*—H···*A*
O1—H15···N4	0.85	2.08	2.929 (3)	178
O1—H16···O2^iii^	0.85	1.87	2.717 (2)	177
O2—H17···N2^i^	0.85	2.08	2.923 (2)	170
O2—H18···O1	0.85	1.89	2.733 (2)	172

Symmetry codes: (iii) *x*−1, *y*, *z*; (i) −*x*+2, −*y*+1, −*z*+1.
